# Meta-analysis of randomised adjuvant therapy trials for pancreatic cancer

**DOI:** 10.1038/sj.bjc.6602513

**Published:** 2005-04-05

**Authors:** D D Stocken, M W Büchler, C Dervenis, C Bassi, H Jeekel, J H G Klinkenbijl, K E Bakkevold, T Takada, H Amano, J P Neoptolemos

**Affiliations:** 1 Cancer Research UK Clinical Trials Unit, University of Birmingham, Birmingham, UK; 2 University of Heidelberg, Heidelberg, Germany; 3 Agia Olga Hospital, Athens, Greece; 4 University of Verona, Verona, Italy; 5 University Hospital Rotterdam, Rotterdam, The Netherlands; 6 University of Bergen, Bergen, Norway; 7 Teikyo University School of Medicine, Teikyo, Japan; 8 University of Liverpool, Liverpool, UK

**Keywords:** pancreas, resection, post-operative, chemotherapy, chemoradiation, radiotherapy

## Abstract

The aim of this study was to investigate the worldwide evidence of the roles of adjuvant chemoradiation and adjuvant chemotherapy on survival in potentially curative resected pancreatic cancer. Five randomised controlled trials of adjuvant treatment in patients with histologically proven pancreatic ductal adenocarcinoma were identified, of which the four most recent trials provided individual patient data (875 patients). This meta-analysis includes previously unpublished follow-up data on 261 patients. The pooled estimate of the hazard ratio (HR) indicated a 25% significant reduction in the risk of death with chemotherapy (HR=0.75, 95% confidence interval (CI): 0.64, 0.90, *P*-values_stratified_ (*P*_strat_)=0.001) with median survival estimated at 19.0 (95% CI: 16.4, 21.1) months with chemotherapy and 13.5 (95% CI: 12.2, 15.8) without. The 2- and 5-year survival rates were estimated at 38 and 19%, respectively, with chemotherapy and 28 and 12% without. The pooled estimate of the HR indicated no significant difference in the risk of death with chemoradiation (HR=1.09, 95% CI: 0.89, 1.32, *P*_strat_=0.43) with median survivals estimated at 15.8 (95% CI: 13.9, 18.1) months with chemoradiation and 15.2 (95% CI: 13.1, 18.2) without. The 2- and 5-year survival rates were estimated at 30 and 12%, respectively, with chemoradiation and 34 and 17% without. Subgroup analyses estimated that chemoradiation was more effective and chemotherapy less effective in patients with positive resection margins. These results show that chemotherapy is effective adjuvant treatment in pancreatic cancer but not chemoradiation. Further studies with chemoradiation are warranted in patients with positive resection margins, as chemotherapy appeared relatively ineffective in this patient subgroup.

Pancreatic ductal adenocarcinoma is one of the top five causes of cancer death in the Western world (Bramhall *et al*, 1995; Parkin *et al*, 2001). Long-term survival remains poor with a 5-year survival rate of 0.4% (Bramhall *et al*, 1995) to 4% (Jemal *et al*, 2003). Resection is associated with improved survival, but this is only possible in approximately 10% of patients (Sener *et al*, 1999). Postoperative adjuvant therapy may further improve long-term survival, but its routine use has yet to be properly established (Neoptolemos *et al*, 2003). Adjuvant therapy can be in the form of chemoradiation or chemotherapy or both.

Until the recent European Study Group for Pancreatic Cancer (ESPAC)1 trial, studies of adjuvant therapy have either combined patients with both pancreatic and periampullary cancers, have been underpowered to detect the modest survival differences expected of postoperative adjuvant treatment or have been nonrandomised (Neoptolemos *et al*, 2003). The ESPAC1 trial was designed and powered to answer two questions: the roles of adjuvant chemoradiation and adjuvant chemotherapy, randomising 289 patients into a 2 × 2 factorial design and the recent publication of the final results showed no survival benefit for chemoradiation but a significant survival benefit for chemotherapy (ESPAC1-2 × 2) (Neoptolemos *et al*, 2001a, 2004). The results of individual studies can be conflicting in isolation and the recent results of the ESPAC1 trial have highlighted the need to perform this meta-analysis to assess all available worldwide evidence addressing these questions. In particular, the ESPAC group randomised an additional 261 patients outside of the 2 × 2 factorial design (ESPAC1-plus) (Neoptolemos *et al*, 2001a) and the updated evidence from these patients are also presented here for the first time. Also, combining trial results in this meta-analysis increases statistical power and has allowed reliable assessment of all available randomised evidence (Stewart and Parmar, 1993; Stewart and Clarke, 1995; Clarke and Godwin, 1998; Parmar *et al*, 1998), in particular, the assessment of the magnitude of treatment effects within predefined prognostic subgroups.

This meta-analysis was an international collaboration collating individual patient data from randomised controlled trials (Kalser and Ellenberg, 1985; Gastrointestinal Study Group, 1987; Bakkevold *et al*, 1993; Klinkenbijl *et al*, 1999; Neoptolemos *et al*, 2001a, 2004; Takada *et al*, 2002) with the primary aim to investigate the roles of adjuvant chemoradiation and adjuvant chemotherapy on survival.

## PATIENTS AND METHODS

### Data collection

A protocol for the meta-analysis including objectives, inclusion criteria for trials and data analysis was followed. A systematic literature search, using ISI Web of Knowledge, Medline and EMBASE search tools, was carried out as part of a review (Neoptolemos *et al*, 2003) to identify randomised controlled trials published worldwide. Information regarding ongoing trials was also requested from members of ESPAC group across 11 European countries. Trials were eligible if they randomised patients to adjuvant therapy for resected pancreatic cancer recruiting only patients aged >18 years with histologically proven ductal adenocarcinoma of the pancreas and the trial had closed to recruitment. Identified trial groups were contacted for access to their individual patient data and trial protocols.

Meta-analysis of individual patient data provides increased accuracy (Stewart and Parmar, 1993; Stewart and Clarke, 1995; Clarke and Godwin, 1998; Parmar *et al*, 1998) and as such trial groups were encouraged to supply individual patient data including prognostic information and additional follow-up for survival, where possible. Patient characteristics and baseline histology data were requested including date of birth, sex, date of operation, date of randomisation, tumour type and size, grade of disease, microscopic resection margin status (Neoptolemos *et al*, 2001b), nodal status, smoking and preoperative diabetes status as well as survival information based on the date last seen alive, date and cause of death and randomised treatment group.

Only patients with pancreatic ductal adenocarcinoma were included since the survival rate of other less common types of pancreatic or ‘periampullary’ cancer such as ampullary carcinoma and intrapancreatic bile duct cancer have much more favourable prognoses (Magee *et al*, 2002). Patients were grouped according to the type of randomised adjuvant treatment (chemoradiation or chemotherapy). The treatments investigated by the individual trials were commonly used regimens that were fairly well tolerated by patients and as such reported no significant detriment to quality of life.

### Statistical methods

The main outcome measure for analysis was overall survival measured from date of operation to the date of death (from all causes) or censor date. All analyses were carried out on an intention-to-treat basis in that patients were analysed according to their allocated treatment group irrespective of treatment they received. Any ineligible patients deleted from published analyses were reinstated where data were available. Reanalysis was carried out comparing Kaplan–Meier estimates of survival (Kaplan and Meier, 1958) using standard and stratified log-rank tests (Peto *et al*, 1977). The log-rank expected numbers of deaths and variance were used to calculate individual and pooled hazard ratios (HR) together with confidence intervals (CIs). Hazard ratios indicating the effect of treatment on the risk of death are graphically presented (Early Breast Cancer Trialists Collaborative Group, 1990), with *χ*^2^ tests used to test for statistical heterogeneity (interaction) across trials and across prespecified clinical subgroups of patients.

Resection margin status was recognised as an influential prognostic factor by which to stratify randomisation (Kalser and Ellenberg, 1985; Gastrointestinal Study Group, 1987; Bakkevold *et al*, 1993; Neoptolemos *et al*, 2001a, 2004). Other stratification factors at randomisation were tumour location (head *vs* body/tail; Kalser and Ellenberg, 1985; Gastrointestinal Study Group, 1987), pancreatic *vs* ampullary (Klinkenbijl *et al*, 1999; Neoptolemos *et al*, 2001a, 2004; Takada *et al*, 2002), centre (Klinkenbijl *et al*, 1999; Neoptolemos *et al*, 2001a, 2004; Takada *et al*, 2002), gender (Bakkevold *et al*, 1993) and type of surgery, differentiation and stage of disease (Kalser and Ellenberg, 1985; Gastrointestinal Study Group, 1987). Reported influential prognostic factors for pancreatic adenocarcinoma were performance status and extent of tumour (Kalser and Ellenberg, 1985; Gastrointestinal Study Group, 1987) and tumour grade, regional lymph node status and tumour size (Neoptolemos *et al*, 2001a, 2004). As such, clinical subgroups were specifically those defined by resection margin status (negative, positive), tumour grade (well, moderate, poor differentiation), nodal status (negative, positive), tumour size (<2 cm, >2 cm) and age (<60 years, >60 years). Trials were grouped according to the type of adjuvant treatment used (chemoradiation or chemotherapy).

## RESULTS

### Trials and patients

Searches identified four published studies of adjuvant systemic chemotherapy, 14 published studies of adjuvant external beam radiotherapy and 12 studies of combination adjuvant chemoradiation plus maintenance chemotherapy (Neoptolemos *et al*, 2003). Two of the four adjuvant chemotherapy studies were randomised controlled trials and two were retrospective surveys. Two of the 14 radiotherapy studies were randomised controlled trials, six were nonrandomised and a further six used intraoperative radiotherapy. One of the 12 combination studies was a randomised controlled trial, with the other 11 being nonrandomised or retrospective studies. As such, two adjuvant systemic chemotherapy studies (Bakkevold *et al*, 1993; Neoptolemos *et al*, 2001a, 2004), two adjuvant chemoradiation studies (Klinkenbijl *et al*, 1999; Neoptolemos *et al*, 2001a, 2004) and one study of adjuvant chemoradiation plus maintenance chemotherapy (Kalser and Ellenberg, 1985; Gastrointestinal Study Group, 1987) were prospective, randomised trials identified for inclusion in this meta-analysis. One additional trial of adjuvant systemic chemotherapy (Takada *et al*, 2002) was published following the review article and was eligible for inclusion. The results of a Radiation Therapy Oncology Group (RTOG) trial (Regine, 2001) of adjuvant chemoradiation plus chemotherapy were not available at the time of this meta-analysis.

Five randomised controlled trials were available for inclusion (Table 1) randomising a total of 1386 patients, of whom 939 had pancreatic cancer. One of the adjuvant systemic and adjuvant chemoradiation studies was the ESPAC1 trial using a 2 × 2 factorial design to answer both questions using the same sample of patients. As such, three trials investigated the use of adjuvant chemoradiation as follows: (i) the Gastrointestinal Study Group (GITSG) showed that adjuvant chemoradiotherapy with maintenance chemotherapy may help improve survival (Kalser and Ellenberg, 1985; Gastrointestinal Study Group, 1987); (ii) the European Organisation for Research and Treatment of Cancer (EORTC) showed no significant advantage for adjuvant chemoradiotherapy (Klinkenbijl *et al*, 1999); and (iii) the ESPAC group showed no benefit for adjuvant chemoradiation (Neoptolemos *et al*, 2001a, 2004). The following three trials investigated the use of adjuvant chemotherapy: (i) the Norwegian Pancreatic Trials Group showed an overall significant increase in median survival advantage for chemotherapy but not 5-year survival (Bakkevold *et al*, 1993); (ii) the Japanese Pancreatic Group concluded that combination chemotherapy did not improve survival in pancreatic patients (Takada *et al*, 2002); and (iii) the ESPAC group showed a survival advantage for adjuvant chemotherapy (Neoptolemos *et al*, 2001a, 2004). The ESPAC group also randomised an additional 261 pancreatic cancer patients (ESPAC1-plus) outside of the 2 × 2 factorial design (69 for chemoradiation *vs* observation and 192 for chemotherapy *vs* observation) and the updated evidence from these patients are presented here for the first time as separate study. The interim results of the ESPAC1-2 × 2 and ESPAC1-plus trials were first published together after a median (interquartile range) follow-up of 10 (1, 25) months (Neoptolemos *et al*, 2001a). The final results of the ESPAC1-2 × 2 factorial trial were published separately after a median (interquartile range) follow-up of 47 (33, 62) months (Neoptolemos *et al*, 2004). This meta-analysis includes the updated results of the ESPAC1-plus trial with a median (interquartile range) follow-up of 39.2 (19.4, 63.9) months, and thus contributes wholly original and previously unpublished data.

The GITSG trial was not able to provide individual patient data due to the age of the trial. The four remaining trials supplied data from 875 pancreatic cancer patients, ranging from 47 to 289 patients within individual trials (Table 2). Eligibility criteria required patients to start adjuvant treatment within 8 weeks of surgery. Patient demographics (age and sex) and important tumour characteristics (resection margin and lymph nodal status on pathology) were provided for all studies. Overall, patients were predominantly male (58%) and aged >60 years of age (55%). As expected, the majority of patients had negative resection margins (68%), ranging from 100% in the Norwegian trial to 17% in the Japanese trial, and approximately half (53%) had regional lymph nodal involvement, ranging from 33% in the Norwegian trial to 60% in the Japanese trial. Both the GITSG and Norwegian trials recruited only patients with negative resection margins, but interestingly the Japanese trial recruited more patients with positive resection margins, against the natural distribution of this factor within this disease. Grade of disease was not available for the Norwegian trial and tumour size was unavailable for the GITSG and Japanese trials. Despite this, over half (52%) of the patients had moderately differentiated tumours, with 19% having poorly differentiated tumours and almost three-quarters (74%) had tumours with maximum dimension >2 cm. Postoperative complications were reported in 27% of all patients, ranging from 22 to 38% in individual trials. Survival data were provided for all patients and as expected, the majority of patients (80%) had died with over three-quarters of patients within each trial having died. The median follow-up for alive patients was at least 24 months within individual trials and 44 (interquartile range: 24.9–63.8) months overall.

### Adjuvant chemoradiation

**Figure 1:**
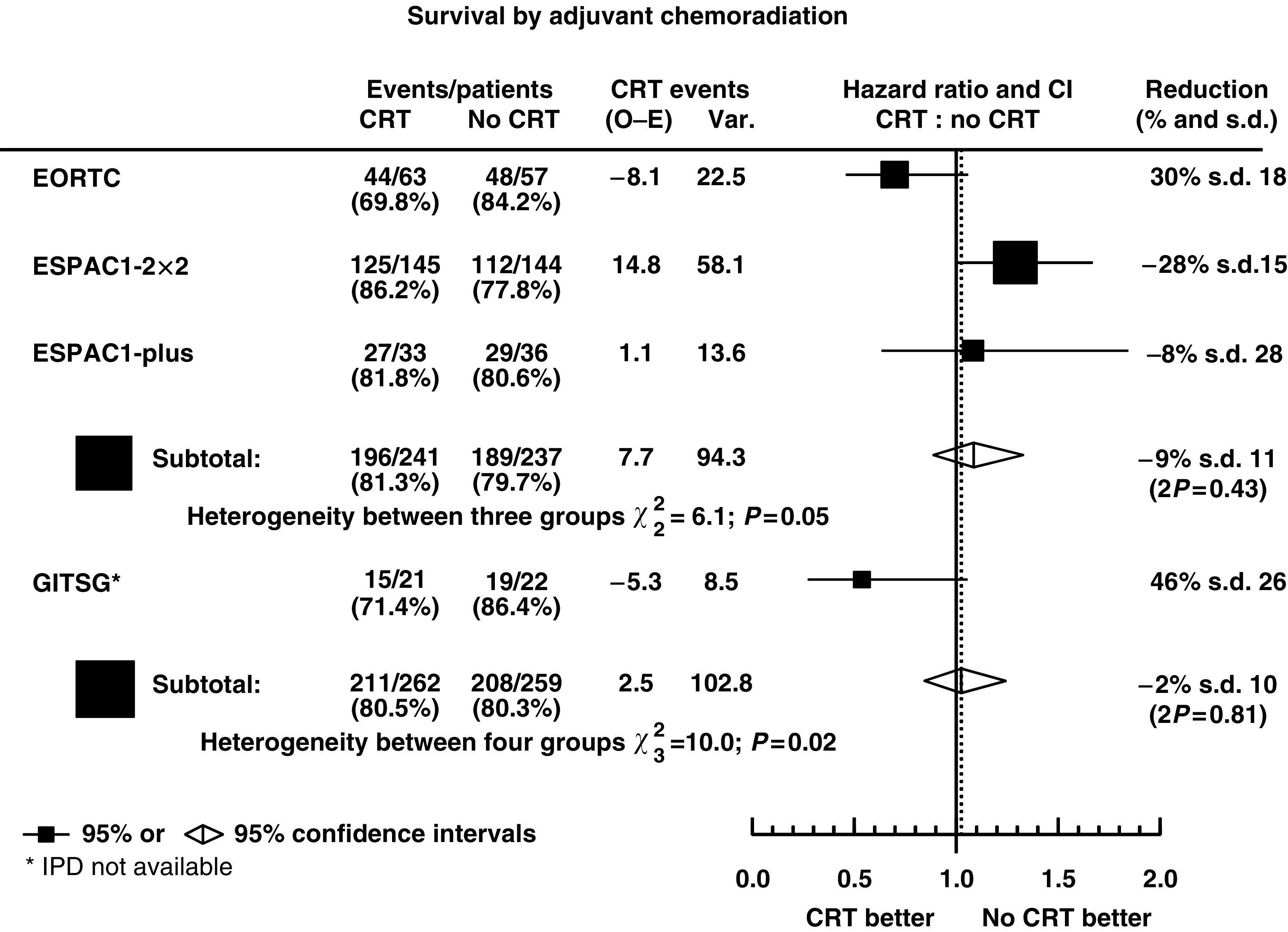
Hazard ratio plot of the effect of chemoradiation in the EORTC, ESPAC1 and GITSG randomised trials (CRT=adjuvant chemoradiation; ▪=individual estimate of the hazard ratio; ⋄=pooled stratified estimate of the hazard ratio).

**Figure 2:**
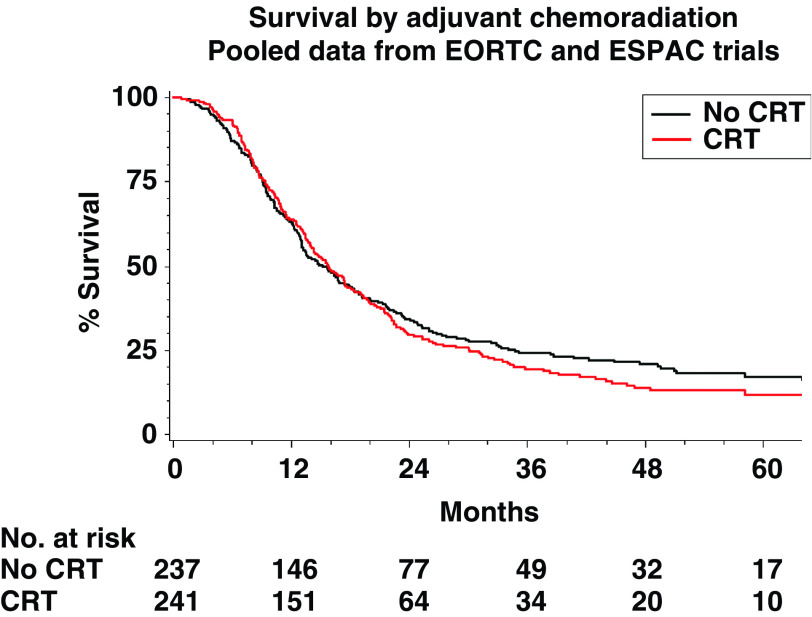
Kaplan–Meier survival estimates by adjuvant chemoradiation in the EORTC and ESPAC1 trials (CRT=adjuvant chemoradiation).

The EORTC (Klinkenbijl *et al*, 1999) and ESPAC (Neoptolemos *et al*, 2001a, 2004) trials were designed to investigate the role of adjuvant chemoradiation using similar schedules of 2 × 20 Gy radiotherapy with 5-fluorouracil (5FU) and folinic acid (FA) as a radiosensitiser randomising a total of 478 patients (385 deaths). Table 3 shows the reanalysis of these trial data and Figure 1 shows the estimates of the HRs of the effect of chemoradiation treatment with 95% CIs. The EORTC trial showed a nonsignificant trend in favour of chemoradiation with a 30% reduction in the risk of death and the ESPAC1-2 × 2 and the ESPAC1-plus trials showed nonsignificant trends in favour of no chemoradiation with 28 and 8% increase in the risk of death, respectively. There was borderline heterogeneity between these results (*χ*^2^=6.1, *P*=0.05), but when combining the trials, the pooled estimate of the HR indicated no significant difference in the risk of death with chemoradiation (HR=1.09, 95% CI: 0.89, 1.32, *P*-values_stratified_ (*P*_strat_)=0.43) being dominated by the ESPAC1 data. The lack of significant benefit for chemoradiation was shown in the survival distributions of patients in the EORTC and ESPAC1 trials combined by treatment (Figure 2) with median survivals estimated at 15.8 (95% CI: 13.9, 18.1) months with chemoradiation and 15.2 (95% CI: 13.1, 18.2) without. The 2- and 5-year survival rates were estimated at 30 and 12%, respectively, with chemoradiation and 34 and 17% without.

Summary statistics estimated from results presented in the GITSG trial paper (Kalser and Ellenberg, 1985) showed a borderline reduction in the risk of death with chemoradiation of 46% (HR=0.54, 95% CI: 0.27, 1.05), more in line with the EORTC results (Table 3). Including these results, using published summary information rather than independent patient data (Figure 1) increased the heterogeneity between the results (*χ*^2^=10.0, *P*=0.02), with the pooled HR again indicating no significant difference in the risk of death with chemoradiation (HR=1.02, 95% CI: 0.85, 1.24, *P*_strat_=0.81).

### Adjuvant chemotherapy

**Figure 3:**
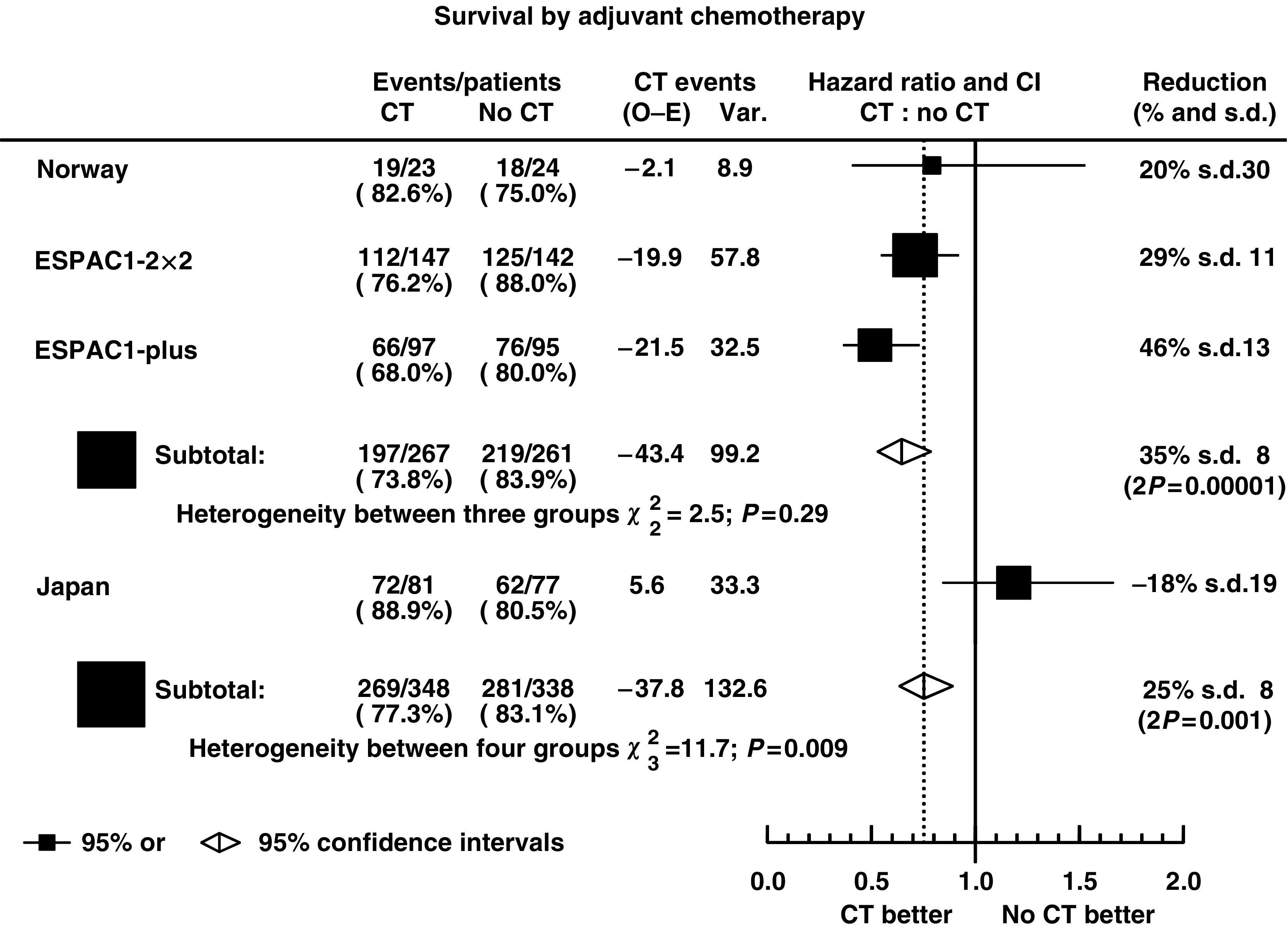
Hazard Ratio plot of the effect of chemotherapy in the Norwegian, ESPAC1 and Japanese trials (CT=adjuvant chemotherapy; ▪=individual estimate of the hazard ratio; ⋄=pooled stratified estimate of the hazard ratio).

**Figure 4:**
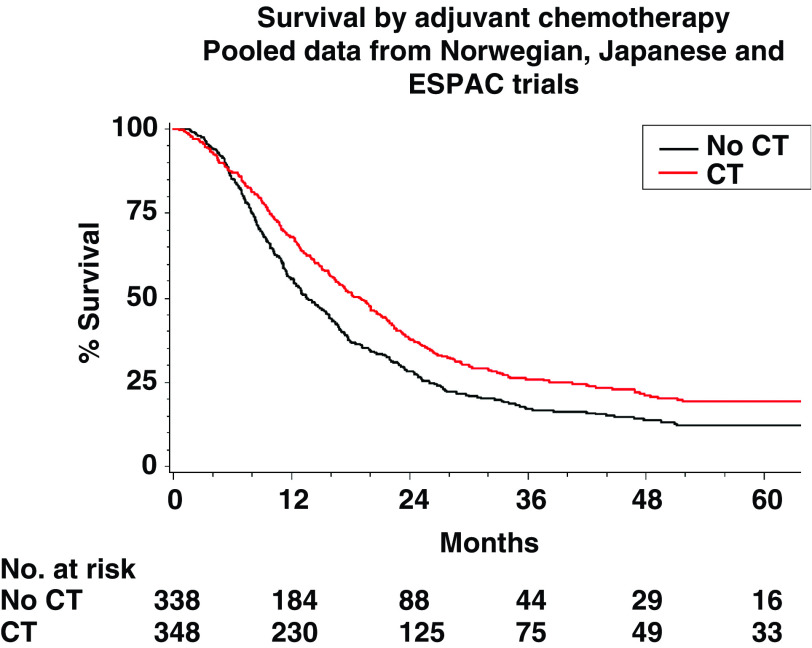
Kaplan–Meier survival estimates by adjuvant chemotherapy in the Norwegian, ESPAC1 and Japanese trials (CT=adjuvant chemotherapy).

The Norwegian (Bakkevold *et al*, 1993), Japanese (Takada *et al*, 2002) and ESPAC (Neoptolemos *et al*, 2001a, 2004) trials were designed to investigate the role of adjuvant chemotherapy using 5FU-based chemotherapy combinations (doxorubicin, mytomycin C and intravenous 5FU combination (Bakkevold *et al*, 1993); mytomycin C, intravenous 5FU and oral 5FU combination (Takada *et al*, 2002); intravenous 5FU and FA combination (Neoptolemos *et al*, 2001a, 2004)) randomising a total of 686 patients (550 deaths). Table 3 shows the reanalysis of these trial data and Figure 3 shows the estimates of the HRs of the effect of chemotherapy treatment with CIs. The Norwegian trial showed a nonsignificant trend in favour of treatment with a 20% reduction in the risk of death, the Japanese trial showed a nonsignificant trend in favour of no treatment with an 18% increase in the risk of death and the ESPAC1-2 × 2 and ESPAC1-plus trials showed a significant trend in favour of chemotherapy with 29 and 46% reduction in the risk of death with chemotherapy, respectively. There was significant heterogeneity between these results (*χ*^2^=11.7, *P*=0.009) due to the inclusion of the Japanese trial, with an unusually high proportion of patients with positive resection margins. When excluding this trial, the heterogeneity between the Norwegian and ESPAC1 studies was markedly reduced (*χ*^2^=2.5, *P*=0.29) to a nonsignificant level. The pooled estimate of the HR, excluding the Japanese trial, indicates a 35% significant reduction in the risk of death with chemotherapy (HR=0.65, 95% CI: 0.54, 0.80, *P*_strat_<0.001) being dominated by the ESPAC1 results. When combining all data including the Japanese trial, the pooled estimate of the HR indicated a 25% significant reduction in the risk of death with chemotherapy (HR=0.75, 95% CI: 0.64, 0.90, *P*_strat_=0.001). The overall benefit for chemotherapy was shown by the survival distributions of patients in the Norwegian, Japanese and ESPAC1 trials combined by treatment (Figure 4) with median survival estimated at 19.0 (95% CI: 16.4, 21.1) months with chemotherapy and 13.5 (95% CI: 12.2, 15.8) without. The 2- and 5-year survival rates were estimated at 38 and 19%, respectively, with chemotherapy and 28 and 12% without.

### Chemoradiation effect within prognostic subgroups

**Figure 5:**
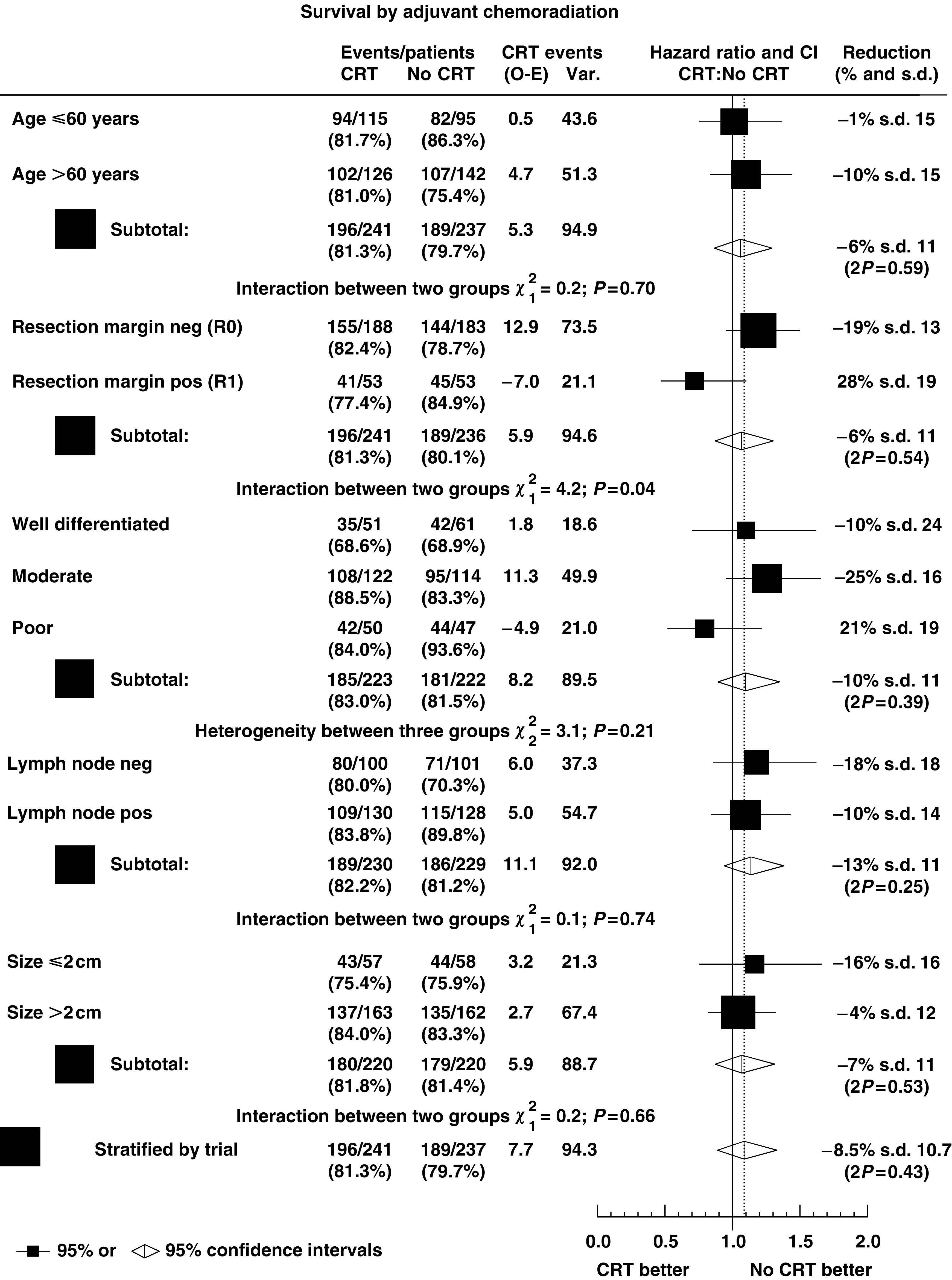
Hazard ratio plot of the effect of chemoradiation by prognostic subgroups in the EORTC and ESPAC1 trials (CRT=adjuvant chemoradiation; ▪=individual estimate of the hazard ratio; ⋄=pooled stratified estimate of the hazard ratio).

Figure 5 shows the estimates of the HRs with CIs of the effect of chemoradiation within the prognostic subgroups, and overall HRs stratified by age, resection margin status, tumour grade, lymph node status and tumour size. The pooled estimates of the HRs stratified by each prognostic factor (*P*_strat_ ranging fro 0.25 to 0.59) confirm the overall lack of benefit seen in the estimate of the HR stratified by trial (*P*_strat_=0.43). There was no significant heterogeneity within the subgroups, except for a significant difference in the effect of chemoradiation dependent upon resection margin status (*χ*^2^=4.2, *P*=0.04), where chemoradiation was estimated to be effective in patients with positive resection margins. However, this effect within this subgroup has a wide CI spanning unity.

### Chemotherapy effect within prognostic subgroups

**Figure 6:**
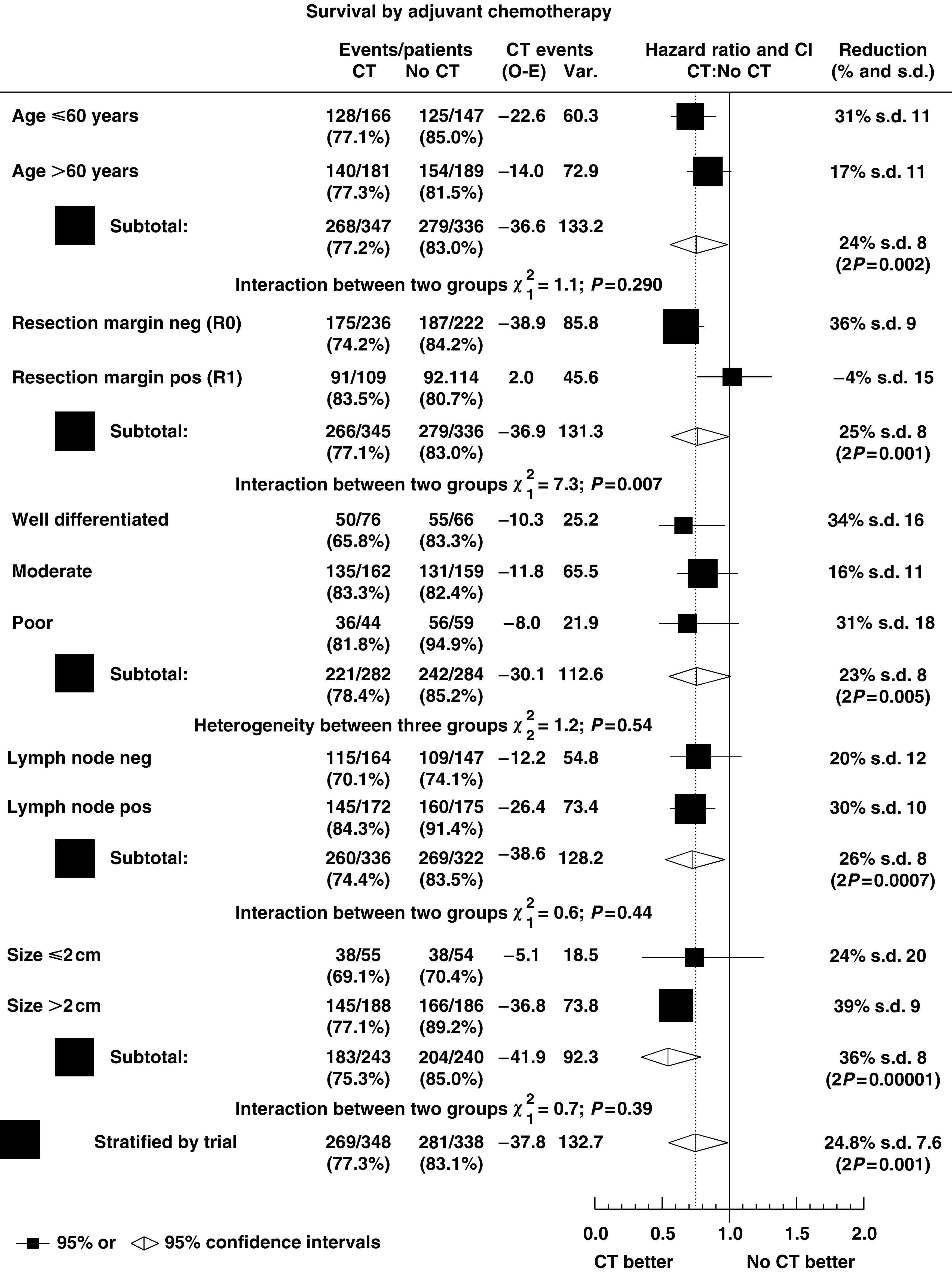
Hazard ratio plot of the effect of chemotherapy by prognostic subgroups in the Norwegian, ESPAC1 and Japanese trials (CT=adjuvant chemotherapy; ▪=individual estimate of the hazard ratio; ⋄=pooled stratified estimate of the hazard ratio).

Figure 6 shows the estimates of the HRs with CIs of the effect of chemotherapy, respectively, within the prognostic subgroups, and overall HRs stratified by age, resection margin status, tumour grade, lymph node status and tumour size. The pooled estimates of the HRs stratified by each prognostic factor confirm the significant decrease in the risk of death with adjuvant chemotherapy (between 23 and 36%, *P*_strat_ <0.005) seen in the estimate of the HR stratified by trial (decreased risk of 25%, *P*_strat_=0.001). There was no significant heterogeneity within the subgroups, except for a significant difference in the effect of chemotherapy dependent upon resection margin status (*χ*^2^=7.3, *P*=0.007), where chemotherapy was estimated to be less effective in patients with positive resection margins.

## DISCUSSION

The recent publication of the ESPAC1 trial results (Neoptolemos *et al*, 2004) highlighted the need to perform this meta-analysis to assess all available worldwide evidence assessing the role of adjuvant treatment following resection of pancreatic cancer. The aim was to provide the most up to date and reliable summary of available evidence of the roles of adjuvant chemoradiation and adjuvant chemotherapy. There have been five published randomised controlled trials (Kalser and Ellenberg, 1985; Gastrointestinal Study Group, 1987; Bakkevold *et al*, 1993; Klinkenbijl *et al*, 1999; Neoptolemos *et al*, 2001a, 2004; Takada *et al*, 2002), of which four provided individual patient data (Bakkevold *et al*, 1993; Klinkenbijl *et al*, 1999; Neoptolemos *et al*, 2001a, 2004; Takada *et al*, 2002). Two trials investigated the role of adjuvant chemotherapy (Bakkevold *et al*, 1993; Takada *et al*, 2002), one investigated the role of adjuvant chemoradiotherapy (Klinkenbijl *et al*, 1999), one investigated both chemoradiotherapy and maintenance chemotherapy (Kalser and Ellenberg, 1985; Gastrointestinal Study Group, 1987) and one trial investigated both (Neoptolemos *et al*, 2001a, 2004). This international collaboration has provided the largest series of randomised individual patient data investigating the use of adjuvant therapy to date, which has allowed particular assessment of the magnitude of any treatment benefit within predefined prognostic subgroups.

The GITSG (Kalser and Ellenberg, 1985; Gastrointestinal Study Group, 1987), EORTC (Klinkenbijl *et al*, 1999) and ESPAC1 (Neoptolemos *et al*, 2001a, 2004) trials were designed to investigate the role of adjuvant chemoradiation randomising a total of 521 patients (419 deaths) and concluded no significant survival benefit with chemoradiation, confirmed within established prognostic factor subgroups. The Norwegian (Bakkevold *et al*, 1993), Japanese (Takada *et al*, 2002) and ESPAC1 (Neoptolemos *et al*, 2001a, 2004) trials were designed to investigate the role of adjuvant chemotherapy using 5FU-based chemotherapy combinations randomising a total of 686 patients (550 deaths) and concluded a significant survival benefit with chemotherapy, confirmed within established prognostic factor subgroups. This is despite the fact that the Japanese trial used a treatment regimen that was largely based on oral 5FU, which because of its hepatic metabolism has very poor efficacy compared to intravenously administered 5FU or specially designed oral fluoropyrimidines (Shore *et al*, 2003).

The assessment of treatment benefit within prespecified prognostic groups is informative for future trial design and patient eligibility. Significant differences in the effect of both chemoradiation and chemotherapy treatments were seen between patients with negative and positive resection margins. Chemoradiation was estimated to be more effective and chemotherapy was estimated to be less effective in patients with positive resection margins; however, neither of these treatment effects was significant within this specific subgroup of patients. The testing of treatments within specific subgroups was purely exploratory, so results should be interpreted with caution due to a lack of statistical power. Nevertheless, this meta-analysis has highlighted the need for further studies to test the effect of treatments, including chemoradiation, specifically in patients with positive resection margins. The ESPAC1-2 × 2 factorial trial (Neoptolemos *et al*, 2004) showed separation of the survival curves in favour of adjuvant chemotherapy commencing at around 8 months but against chemoradiation with the survival curves not beginning to separate until 14 months following resection. Thus, the initial use of chemoradiation appeared to have delayed the effective use of systemic chemotherapy and thereby reduced median and 5-year survival in patients who received the sequential combination. This meta-analysis, however, has pointed to a potential role for chemoradiation, but only in patients with positive resection margins.

There was heterogeneity between trials ascribed to differing patient populations with specific tumour characteristics, specifically the recruitment of resection margin-positive patients. Heterogeneity was influenced by the inclusion of the GITSG (Kalser and Ellenberg, 1985; Gastrointestinal Study Group, 1987) trial, the only trial investigating the effect of chemoradiation in patients with only negative resection margins and the Japanese (Takada *et al*, 2002) trial with an unusually high proportion of patients with positive resection margins. This meta-analysis has been dominated by the evidence from the ESPAC1 (Neoptolemos *et al*, 2001a, 2004) trials. The ESPAC group randomised 289 pancreatic cancer patients for both chemoradiation and chemotherapy treatments as part of a 2 × 2 factorial design and showed a survival advantage for adjuvant chemotherapy but no benefit for adjuvant chemoradiation. The ESPAC group also randomised an additional 261 patients outside of the 2 × 2 factorial design, originally analysed with a median 10 month follow-up and presented here for the first time with a median follow-up of 39.2 months and with similar results.

This meta-analysis provides the most current overview of evidence estimating the effect of adjuvant treatment following ‘curative’ resection of pancreatic cancer, including recent published and unpublished data from the ESPAC1 trial. Pancreatic tumours do not appear to respond well to adjuvant chemoradiation and routine use is not warranted as standard treatment. There may be scope for future studies to investigate more modern chemoradiation techniques including conformal radiotherapy (Regine, 2001) and also further investigation of the potential role for chemoradiation in patients with positive resection margins. There is now strong evidence of a survival benefit for adjuvant chemotherapy and standard care of patients with resectable pancreatic cancer should now be based on curative surgery followed by adjuvant systemic chemotherapy. These results advocate the need for further randomised trials to find the optimal chemotherapy regimen.

## Figures and Tables

**Table 1:** Details of published randomised controlled trials of adjuvant treatment for pancreatic ductal adenocarcinoma

**Trial**	**Recruitment**	**Comparison**	**Adjuvant treatment**	**Number of patients and IPD available**	**Published conclusions**
GITSG^a^, 1985, 1987	Feb '74–May '82 All R0 resections	CRT *vs* OBS	2 × (20 Gy in 10 fractions+500 mg m^−2^ 5FU days 1–3)+weekly 5FU to recurrence	49 pancreatic patients randomized No IPD available	Significant increase in median survival (20 *vs* 11 mo, *P*=0.035) in 43 eligible patients
Norway, 1993	April '84–April '87 All R0 resections	CT *vs* OBS	AMF (40 mg m^−2^ doxorubicin, 6 mg m^−2^ mytomycin C, 500 mg m^−2^ 5FU) once every 3 weeks for six courses	61 patients (47 pancreatic, 14 ampullary) randomised 46 additional nonrandomised patients IPD for 47 pancreatic patients.	Significant increase in median survival (23 *vs* 11 mo, *P*=0.02) in 60 pancreatic and ampullary patients combined
EORTC, 1999	Sept '87–April '95 R0+R1 resections	CRT *vs* OBS	2 × (20 Gy in 10 fractions+25 mg kg^−1^ 5FU/FA days 1–5)	218 patients (120 pancreatic, 98 ampullary) randomised IPD for 120 pancreatic patients	NS increase in median survival (25 *vs* 19 mo, *P*=0.21) in 207 eligible patients NS increase in median survival in 114 eligible pancreatic (17 *vs* 13 mo, *P*=0.099)
Japan, 2002	April '86–June '92 R0−R3 resections	CT *vs* OBS	6 mg m^−2^ mytomycin C day 1+310 mg m^−2^ 5FU days 1–5 and days 15–20 followed by 100 mg m^−2^ oral 5FU daily to recurrence	508 patients (173 pancreatic, 335 bile duct/gallbladder/ampullary) randomised IPD for 158 eligible pancreatic patients	Significant survival benefit in gallbladder No difference in 158 eligible pancreatic No difference in 48 eligible ampullary
ESPAC1-2 × 2, 2001, 2004	Feb '94–June 2000 R0+R1 resections	CRT *vs* no CRT CT *vs* no CT	2 × (20 Gy in 10 fractions+500 mg m^−2^ 5FU/FA days 1–3) (20 mg m^−2^ FA+425 mg m^−2^ 5FU days 1–5) × six cycles	289 pancreatic patients randomised IPD for 289 pancreatic patients	NS decrease in survival for CRT (*P*=0.053) in 289 patients Significant increase in survival for CT (*P*=0.009) in 289 eligible patients
ESPAC1-plus, 2001, updated (unpublished)	Feb '94–June 2000 R0+R1 resections	CRT *vs* no CRT CT *vs* no CT	2 × (20 Gy in 10 fractions+500 mg m^−2^ 5FU/FA days 1–3) (20 mg m^−2^ FA+425 mg m^−2^ 5FU days 1–5) × six cycles	261 pancreatic patients randomised (69 for CRT, 192 for CT) IPD for 261 pancreatic patients	NS decrease in survival for CRT (*P*=0.078) in 69 patients Overall significant increase in survival for CT (*P*<0.001) in 192 patients
Total				1386 patients randomised 939 pancreatic patients randomised IPD for 875 pancreatic patients	

GITSG=Gastrointestinal Study Group; EORTC=European Organisation for Research and Treatment of Cancer; ESPAC=European Study Group for Pancreatic Cancer; R0=resection margin negative; R1=resection margin positive; CRT=adjuvant chemoradiation; CT=adjuvant chemotherapy; OBS=surgery alone; 5FU=5-fluorouracil; FA=folinic acid; NS=nonsignificant.

a Individual Patient Data (IPD) not available.

**Table 2:** Patient characteristics in randomised controlled trials of adjuvant treatment for pancreatic ductal adenocarcinoma

	**Trial**						
**Characteristic**	**GITSG**, ***N*=43**	**Norway**, ***N*=47**	**EORTC**, ***N*=120**	**Japan**, ***N*=158**	**ESPAC1-2 × 2**, ***N*=289**	**ESPAC1-plus**, ***N*=261**	**Total^a^**, ***N*=875**
*Sex*							
Male	26 (61%)	26 (55%)	65 (54%)	93 (59%)	170 (59%)	157 (60%)	511 (58%)
Female	17 (39%)	21 (45%)	55 (46%)	65 (41%)	119 (41%)	104 (40%)	364 (42%)

*Age (years)*							
⩽60	18 (41%)	15 (33%)	57 (47%)	62 (40%)	128 (44%)	133 (51%)	395 (45%)
>60	25 (59%)	31 (67%)	63 (53%)	94 (60%)	161 (56%)	128 (49%)	477 (55%)

*Resection margins*							
Negative (R0)	43 (100%)	47 (100%)	79 (66%)	26 (17%)	238 (82%)	201 (77%)	591 (68%)
Positive (R1)	—	—	40 (34%)	127 (83%)	51 (18%)	60 (23%)	278 (32%)

*Tumour grade*							
Well differentiated	15 (35%)	NA	39 (33%)	43 (29%)	62 (24%)	48 (19%)	192 (25%)
Moderate	26 (60%)		45 (38%)	64 (43%)	148 (56%)	152 (61%)	409 (52%)
Poor	2 (5%)		34 (28%)	12 (8%)	52 (20%)	50 (20%)	148 (19%)
Papillary	—		0	14 (9%)	—	—	14 (2%)
Other	—		1 (1%)	16 (11%)	—	—	17 (2%)

*Lymph nodal status*							
Negative	31 (72%)	31 (67%)	54 (45%)	62 (40%)	119 (43%)	127 (51%)	393 (47%)
Positive	12 (28%)	15 (33%)	66 (55%)	92 (60%)	155 (57%)	122 (49%)	450 (53%)

*Max tumour size (cm)*							
⩽2	NA	13 (28%)	47 (39%)	NA	53 (20%)	58 (25%)	171 (26%)
>2		33 (72%)	72 (61%)		208 (80%)	178 (75%)	491 (74%)

*Postop complication*							
No	NA	29 (62%)	NA	124 (78%)	201 (74%)	177 (72%)	531 (73%)
Yes		18 (38%)		34 (22%)	70 (26%)	70 (28%)	192 (27%)

*Status*							
Alive	9 (21%)	10 (21%)	28 (23%)	24 (15%)	52 (18%)	63 (24%)	177 (20%)
Dead	34 (79%)	37 (79%)	92 (77%)	134 (85%)	237 (82%)	198 (76%)	698 (80%)

*Follow-up of alive patients*							
Median (months)	>12	24.2	33.1	66.8	46.7	39.2	44.1
Interquartile range	—	15.9–31.4	18.9–55.5	48.1–85.0	33.0–62.0	19.4–63.9	24.9–63.8
Range		10.9–46.2	9.4–85.3	1.7–105.9	0.0–93.5	0.4–98.0	0.0–105.9

GITSG=Gastrointestinal Study Group; EORTC=European Organisation for Research and Treatment of Cancer; ESPAC=European Study Group for Pancreatic Cancer; R0=resection margin negative; NA=not available.

a Total excludes the GITSG trial with no individual patient data (IPD).

**Table 3:** Reanalysis of survival data in randomised controlled trials of adjuvant treatment for pancreatic ductal adenocarcinoma

**Trial**	**No. of** **patients**	**No. of** **deaths**	**Median survival** **in months** **(95% CI)**	**2-Year survival rate** **(%)**	**HR (95% CI)**	**Log-rank *χ*^2^**, **significance**
*Chemoradiation (CRT) question*						
GITSG^a^						
No CRT	22	19	10.9 (NA)	15		*P*=0.035
CRT	21	15	20.0 (NA)	42	0.54 (0.27, 1.05)^a^	(one-sided)
EORTC						
No CRT	57	48	12.6 (10.3, 16.3)	23.20		
CRT	63	44	17.5 (13.3, 23.8)	35.70	0.70 (0.46, 1.06)	2.91, *P*=0.088
ESPAC1-2 × 2						
No CRT	144	112	17.9 (14.8, 23.6)	41.40		
CRT	145	125	15.9 (13.7, 19.9)	28.50	1.28 (0.99, 1.66)	3.75, *P*=0.053
ESPAC1-plus						
No CRT	36	29	13.0 (11.5, 15.7)	23.50		
CRT	33	27	12.5 (9.4, 16.6)	24.60	1.08 (0.64, 1.83)	0.09, *P*=0.77

*Chemotherapy (CT) question*						
Norway						
No CT	24	18	10.4 (6.6, 13.1)	24.30		
CT	23	19	17.7 (11.0, 25.6)	30.60	0.80 (0.42, 1.53)	0.49, *P*=0.48
Japan						
No CT	77	62	12.4 (10.5, 19.0)	29.60		
CT	81	72	12.8 (9.8, 16.8)	24.20	1.18 (0.84, 1.66)	0.95, *P*=0.33
ESPAC1-2 × 2						
No CT	142	125	15.5 (13.0, 17.7)	30.00		
CT	147	112	20.1 (16.5, 22.7)	39.70	0.71 (0.55, 0.92)	6.82, *P*=0.009
ESPAC1-plus						
No CT	95	76	12.7 (10.2, 16.6)	26.80		
CT	97	66	24.0 (18.8, 29.4)	48.90	0.54 (0.39, 0.76)	14.19, *P*<0.001

CI=confidence interval; HR=hazard ratio; GITSG=Gastrointestinal Study Group; EORTC=European Organisation for Research and Treatment of Cancer; ESPAC=European Study Group for Pancreatic Cancer; CRT=adjuvant chemoradiation; CT=adjuvant chemotherapy; NA=not available.

a Individual patient data (IPD) not available – data extracted from summary statistics provided in publication, HR estimated from data provided.

## Appendix A

### PANCREATIC META-ANALYSIS GROUP

*European Study Group for Pancreatic Cancer (ESPAC)*: C Bassi, H Beger, MW Büchler, G Butturini, C Dervenis, JA Dunn, M Falconi, L Fernandez-Cruz, H Friess, H Hickey, DJ Kerr, F Lacaine, JP Neoptolemos, A Pap, D Spooner, DD Stocken.

*European Organisation for Research and Treatment of Cancer (EORTC)*: JP Arnaud, L Collette, ML Couvreur, LT De Wit, DG Gonzalez, A Hennipman, J Jeekel, JHG Klinkenbijl, T Sahmoud, R Van Pel, CH Veenhof, J Wils.

*Norwegian Pancreatic Group*: B Arnesjø, KE Bakkevold, O Dahl, B Kambestad.

*Japanese Pancreatic Group*: H Amano, H Kato, T Matsushiro, T Nagakawa, T Nakayama, Y Nimura, T Takada, H Yasuda.

## References

[bib1] Bakkevold KE, Arnesjo B, Dahl O, Kambestad B (1993) Adjuvant combination chemotherapy (AMF) following radical resection of carcinoma of the pancreas and papilla of Vater. Results of a controlled, prospective, randomised multicentre study. Eur J Cancer 29: 698–703 10.1016/s0959-8049(05)80349-1 8471327

[bib2] Bramhall SR, Allum WH, Jones AG, Allwood A, Cummins C, Neoptolemos JP (1995) Incidence, treatment and survival in 13, 560 patients with pancreatic cancer: an epidemiological study in the West Midlands. Br J Surg 82: 111–115 7881926 10.1002/bjs.1800820137

[bib3] Clarke M, Godwin J (1998) Systematic reviews using individual patient data: a map for the minefields? Ann Oncol 9: 827–833 9789604 10.1023/a:1008468705492

[bib4] Early Breast Cancer Trialists' Collaborative Group (1990) Introduction and Methods Sections Reproduced From: Treatment of Early Breast Cancer. Worldwide Evidence 1985–1990 Vol 1, Oxford: Oxford University Press

[bib5] Gastrointestinal Tumor Study Group (1987) Further evidence of effective adjuvant combined radiation and chemotherapy following curative resection of pancreatic cancer. Cancer 59: 2006–2010 3567862 10.1002/1097-0142(19870615)59:12<2006::aid-cncr2820591206>3.0.co;2-b

[bib6] Jemal A, Murray T, Samnels A, Ghafoor A, Ward E, Thun M (2003) Cancer statistics, 2003. CA Cancer J Clin 53: 5–26 12568441 10.3322/canjclin.53.1.5

[bib7] Kalser MH, Ellenberg SS (1985) Pancreatic cancer. Adjuvant combined radiation and chemotherapy following curative resection. Arch Surg 120: 899–903 4015380 10.1001/archsurg.1985.01390320023003

[bib8] Kaplan EL, Meier P (1958) Non parametric estimation from incomplete observations. J Am Stat Assoc 53: 457–481

[bib9] Klinkenbijl JH, Jeekel J, Sahmoud T, van Pel R, Couvreur ML, Veenhof CH, Arnaud JP, Gonzalez DG, de Wit LT, Hennipman A, Wils J (1999) Adjuvant radiotherapy and 5-fluorouracil after curative resection of cancer of the pancreas and periampullary region. Phase III trial of the EORTC GITCCG. Ann Surg 230: 776–784 10615932 10.1097/00000658-199912000-00006 PMC1420941

[bib10] Magee CJ, Ghaneh P, Neoptolemos JP (2002) Surgical and medical therapy for pancreatic carcinoma. Best Pract Res Clin Gastroenterol 16: 435–455 12079268 10.1053/bega.2002.0317

[bib11] Neoptolemos JP, Cunningham D, Friess H, Bassi C, Stocken DD, Tait DM, Dunn JA, Dervenis C, Lacaine F, Hickey H, Raraty MGT, Ghaneh P, Buchler MW (2003) Adjuvant therapy in pancreatic cancer: historical and current perspectives. Ann Oncol 14: 675–692 12702520 10.1093/annonc/mdg207

[bib12] Neoptolemos JP, Dunn JA, Stocken DD, Almond J, Link K, Beger H, Bassi C, Falconi M, Pederzoli P, Dervenis C, Fernandez-Cruz L, Lacaine F, Pap A, Spooner D, Kerr DJ, Freiss H, Büchler MW (2001a) Adjuvant chemoradiotherapy and chemotherapy in resectable pancreatic cancer: a randomised controlled trial. Lancet 358(9293): 1576–1585 11716884 10.1016/s0140-6736(01)06651-x

[bib13] Neoptolemos JP, Stocken DD, Dunn JA, Almond J, Beger HG, Pederzoli P, Bassi C, Dervernis C, Fernandez-Cruz L, Lacaine F, Buckels J, Deakin M, Adab F, Sutton R, Imrie C, Ihse I, Tihanyi T, Olah A, Pedrazzoli S, Spooner D, Kerr DJ, Freiss H, Büchler MW (2001b) Influence of Resection margins on survival for patients with pancreatic cancer treated by adjuvant chemoradiation and/or chemotherapy in the ESPAC-1 randomized controlled trial. Ann Surg 234: 758–768 11729382 10.1097/00000658-200112000-00007 PMC1422135

[bib14] Neoptolemos JP, Stocken DD, Friess H, Bassi C, Dunn JA, Hickey H, Beger H, Fernandez-Cruz L, Dervenis C, Lacaine F, Falconi M, Pederzoli P, Pap A, Spooner D, Kerr DJ, Büchler MW (2004) A randomized trial of chemoradiotherapy and chemotherapy after resection of pancreatic cancer. N Engl J Med 350(12): 1200–1210 15028824 10.1056/NEJMoa032295

[bib15] Parkin DM, Bray FI, Devesa SS (2001) Cancer burden in the year 2000. The global picture. Eur J Cancer 37: S4–S66 11602373 10.1016/s0959-8049(01)00267-2

[bib16] Parmar MKB, Torri V, Stewart L (1998) Extracting summary statistics to perform meta-analyses of the published literature for survival endpoints. Stat Med 17: 2815–2834 9921604 10.1002/(sici)1097-0258(19981230)17:24<2815::aid-sim110>3.0.co;2-8

[bib17] Peto R, Pike MC, Armitage P, Breslow NE, Cox DR, Howard SV, Mantel N, McPherson K, Peto J, Smith PG (1977) Design and analysis of randomised clinical trials requiring prolonged observation of each patient II. Analysis and example. Br J Cancer 35: 1–39 831755 10.1038/bjc.1977.1 PMC2025310

[bib18] Regine WF (2001) Postoperative adjuvant therapy: past, present and future trial development. In MD Anderson Solid Tumor Oncology Series Evans DB, Pisters PWT, Abbruzzese JL, Pollock RE (eds) pp 235–242. New York, USA: Springer-Verlag

[bib19] Sener S, Fremgen A, Menck H, Winchester D (1999) Pancreatic cancer: a report of treatment and survival trends for 100, 313 patients diagnosed from 1985–1995, using the National Cancer database. J Am Coll Surg 189: 1–7 10401733 10.1016/s1072-7515(99)00075-7

[bib20] Shore S, Raraty M, Ghaneh P, Neoptolemos JP (2003) Chemotherapy for pancreatic cancer. Aliment Pharmacol Therap 18: 1049–1069 14653825 10.1111/j.1365-2036.2003.01781.x

[bib21] Stewart LA, Clarke MJ (1995) on behalf of the Cochrane Working Party group on Meta-analysis using Individual Patient Data. Practical methodology of meta-analyses (overviews) using updated individual patient data. Stat Med 14: 2057–2079 8552887 10.1002/sim.4780141902

[bib22] Stewart LA, Parmar MKB (1993) Meta-analysis. Lancet 341: 964

[bib23] Takada T, Amano H, Yasuda H, Nimura Y, Matsushiro T, Kato H, Nagakawa T, Nakayama T (2002) Is postoperative adjuvant chemotherapy useful for gall-bladder carcinoma? Cancer 95(8): 1685 12365016 10.1002/cncr.10831

